# Supplementary material for: Health workers’ perspectives on asthma care coordination between primary and specialised healthcare in the COVID-19 pandemic: a protocol for a qualitative study in Ecuador and Brazil
Source: BMJ Open. 2021 Nov 1;11(11):e052971. doi: 10.1136/bmjopen-2021-052971 (PMC8561830; doi:10.1136/bmjopen-2021-052971)
Supplement: Author's manuscript [file bmjopen-2021-052971.draft_revisions.pdf]

BMJ Open is committed to open peer review. As part of this commitment we make the peer review history of every article we publish publicly available.

When an article is published we post the peer reviewers' comments and the authors' responses online. We also post the versions of the paper that were used during peer review. These are the versions that the peer review comments apply to.

The versions of the paper that follow are the versions that were submitted during the peer review process. They are not the versions of record or the final published versions. They should not be cited or distributed as the published version of this manuscript.

BMJ Open is an open access journal and the full, final, typeset and author-corrected version of record of the manuscript is available on our site with no access controls, subscription charges or pay-per-view fees (http://bmjopen.bmj.com).

If you have any questions on BMJ Open's open peer review process please email info.bmjopen@bmj.com

Health worker's perspectives on asthma care coordination between primary and specialized health care in the COVID-19 pandemic: a protocol qualitative study in Ecuador and Brazil

Journal:	*BMJ Open*
Manuscript ID	bmjopen-2021-052971
Article Type:	Protocol
Date Submitted by the Author:	01-May-2021

Complete List of Authors:	Romero, Natalia; Universidad Internacional del Ecuador, School of Medicine; Red Grups d'Amèrica i Àfrica Llatines<br>Cisneros-Caceres, Maria; Universidad Internacional del Ecuador, School of Medicine<br>Granadillo, Emily; Universidad Internacional del Ecuador, School of Medicine<br>Aragao, Erika; Universidade Federal da Bahia<br>Romero-Sandoval, Adriana; Universidad Internacional del Ecuador, School of Basic Sciences; Red Grups d'Amèrica i Àfrica Llatines<br>Barbosa, Carolina; Universidade Federal da Bahia<br>Barreto de Oliveira, Ana Luiza; Universidade Federal da Bahia<br>Rodriguez, Alejandro ; Universidad Internacional del Ecuador, School of Medicine<br>Pinheiro, Gabriela; Fundação ProAR,<br>Cruz, Alvaro; Universidade Federal da Bahia<br>Cooper, Philip; St George's University of London, Institute of Infection and Immunity; Universidad Internacional del Ecuador, School of Medicine<br>Ferreira da Silva, Maria Rejane; UPE; Red Grups d'Amèrica i Àfrica Llatines

Keywords:	Asthma < THORACIC MEDICINE, COVID-19, Quality in health care < HEALTH SERVICES ADMINISTRATION & MANAGEMENT, HEALTH SERVICES ADMINISTRATION & MANAGEMENT, Organisation of health services < HEALTH SERVICES ADMINISTRATION & MANAGEMENT

SCHOLARONE™
Manuscripts

I, the Submitting Author has the right to grant and does grant on behalf of all authors of the Work (as defined in the below author licence), an exclusive licence and/or a non-exclusive licence for contributions from authors who are: i) UK Crown employees; ii) where BMJ has agreed a CC-BY licence shall apply, and/or iii) in accordance with the terms applicable for US Federal Government officers or employees acting as part of their official duties; on a worldwide, perpetual, irrevocable, royalty-free basis to BMJ Publishing Group Ltd ("BMJ") its licensees and where the relevant Journal is co-owned by BMJ to the co-owners of the Journal, to publish the Work in this journal and any other BMJ products and to exploit all rights, as set out in our licence.

The Submitting Author accepts and understands that any supply made under these terms is made by BMJ to the Submitting Author unless you are acting as an employee on behalf of your employer or a postgraduate student of an affiliated institution which is paying any applicable article publishing charge ("APC") for Open Access articles. Where the Submitting Author wishes to make the Work available on an Open Access basis (and intends to pay the relevant APC), the terms of reuse of such Open Access shall be governed by a Creative Commons licence – details of these licences and which Creative Commons licence will apply to this Work are set out in our licence referred to above.

Other than as permitted in any relevant BMJ Author's Self Archiving Policies, I confirm this Work has not been accepted for publication elsewhere, is not being considered for publication elsewhere and does not duplicate material already published. I confirm all authors consent to publication of this Work and authorise the granting of this licence.

For peer review only

Title: Health worker's perspectives on asthma care coordination between primary and specialized health care in the COVID-19 pandemic: a protocol qualitative study in Ecuador and Brazil

Natalia Romero-Sandoval[1,7] María José Cisneros-Caceres[1], Emily Granadillo[1], Erika Aragao[2], Adriana Romero-Sandoval[3,7], Carolina Barbosa[4], Ana Luiza Barreto de Oliveira[4], Alejandro Rodríguez[1], Gabriela Pimentel-Pinheiro[4], Álvaro A. Cruz[4], Philip Cooper[1,5], Maria Rejane Ferreira da Silva[6 7]

[1]. School of Medicine, Universidad Internacional del Ecuador, Quito, Ecuador

[2]. Instituto de Saúde Coletiva - Universidade Federal da Bahia, Salvador de Bahia – Brazil

[3]. School of Basic Sciences, Universidad Internacional del Ecuador, Quito, Ecuador

[4]. Programa para o Controle da Asma na Bahia (ProAr), Faculdade de Medicina, Universidade Federal da Bahia, Salvador de Bahia – Brazil

[5]. Institute of Infection and Immunity, St George's University of London, London, UK.

[6].Universidade de Pernambuco/UPE, Pernambuco – Brazil

[7]. Red Grups d'Amèrica i Àfrica Llatines – GRAAL

NRS nromero@uide.edu.ec
MJC majocc93@gmail.com
EG egranadillo@uide.edu.ec
ARS adromero@uide.edu.ec
EA erikapecs@gmail.com
CB carolinabarbosa.ss@gmail.com
ALB annabarreto12@yahoo.com.br
GPP gabrielacear@gmail.com
AR rorodriguezal@uide.edu.ec
AAC cruz.proar@gmail.com
PC pcooper@sgul.ac.uk
MRF rejane.pernambuco@gmail.com

For peer review only

Corresponding author: Maria Jose Cisneros-Caceres. Av. Jorge Fernández and Simón Bolívar, Quito, Ecuador. Telf: + 593 2985600

For peer review only

ABSTRACT

Introduction. Asthma is a common long-term disorder and strategies to improve asthma control are still a challenge. Integrated delivery of health systems is critical for effective asthma care: there is limited information on experiences of care coordination for asthma from Latin American, especially on perspectives of health personnel and in the context of the COVID-19 pandemic.

Methods and analysis. This protocol details a qualitative approach to analyse health workers' perspectives of health care coordination for asthma control during COVID-19 pandemic in Ecuador and Brazil, at primary and specialized levels, through in-depth semi-structured interviews using a video communications platform. The analysis will identify knowledge and perspectives based on coordination of clinical information, clinical management, and administrative coordination. Theoretical sampling will be used to obtain approximately equal numbers of women and men within each level of healthcare; data saturation will be used to determine sample size. Transcripts will be analysed using content coding procedures to mark quotations related to major topics and sub-themes included in the interview guide, and narrative analysis will be based on a theoretical framework for health care coordination to identify new themes and sub-themes.

Ethics and dissemination. Ethical approval was obtained from the ethics committees of Hospital General Docente Calderón, Quito, Ecuador, and Universidade Federal da Bahia, Salvador, Brazil. The findings of this study will be disseminated through peer–reviewed articles, conference presentations and condensed summaries for key stakeholders and partners.

Keywords: *Asthma, COVID-19, Quality in health care, Organisation of health services, Health services administration & management*

For peer review only

ARTICLE SUMMARY

Strengths and limitations of this study

- This qualitative study protocol is the first to focus on the perspectives of healthcare workers on asthma care coordination between primary and specialized levels in the context of the COVID-19 pandemic.
- This study will use in-depth semi-structured interviews of healthcare workers from four cities in Ecuador and Brazil to compare perspectives on asthma care and healthcare system coordination between locations.
- Qualitative interviews will be done over the period of a year during the COVID-19 pandemic during that likely will affect coordination of asthma care.
- Although our recruitment method should identify key informants, we could not involve the best representatives from primary and specialized care levels.

For peer review only

INTRODUCTION

Asthma has emerged as a major challenge for health care systems around the world accounting for millions of doctor visits, hundreds of thousands of emergency department visits and hospitalizations, and thousands of deaths (1,2). Among those who have asthma, children are the most affected group and asthma is now the most common chronic disease of childhood (3). While our knowledge on asthma has increased, we still have an incomplete understanding of its causes: factors including environmental exposures, changes in lifestyles and host genetics are likely to be important (1).

There are wide variations in the prevalence of asthma symptoms between countries (4). However, trends in asthma mortality and hospitalization have shown a progressive reduction or stabilization, especially in high income countries (HICs), where asthma prevalence reached epidemic levels four decades ago (5). Presently, although the prevalence of asthma has stabilized in HICs, it appears to be increasing in some low and middle-income countries (LMICs), regions that account for more than 80% of asthma deaths worldwide. Nonetheless, many cases of severe asthma and asthma deaths are preventable through optimal management using medications to relieve and control the disease and improvements in health care coordination (HCC) (1,6).

HCC has been shown to be a promising strategy to improve asthma management and control in HICs: improved care coordination results in reduced asthma symptoms and urgent health care utilization which is cost-effective (7). However, in LMICs health care coordination remains a challenge for asthma patients because most of the health systems in these regions are characterized by fragmentary organization and a lack of resources (8). Additionally, medical therapy is often hindered by fragmented disease-specific approaches to management (fostered by single-disease guidelines), use of acute care to manage chronic diseases, and inadequate integration of care across multiple levels, especially from hospital to home (9,10).

In Latin America (LA), HCC has been characterized by a reconstruction of the self-identity concept (11). There are few reports of HCC implementation (12,13), while asthma management and control are uncoordinated in most health systems, showing high rates of acute exacerbations, and use of urgent care with high hospitalization rates (14,15).

For peer review only

The outbreak of infections with the novel coronavirus, SARS-CoV-2, in Central China, in December 2019 marked the beginning of the COVID-19 pandemic (16). Measures to reduce viral transmission were adopted in Ecuador and Brazil from March 2020 with enforced social isolation and restricted movement at local or national levels interfering with opportunities for interactions with health care. Although lockdowns had been lifted by the second half of 2020, there was still limited access to emergency rooms and outpatient care at primary and specialized levels for non-COVID-19 consults (17), a situation that continued into the 1st quarter of 2021.

This protocol details the methodological and analytical considerations using a qualitative approach in a study designed to understand and characterize healthcare workers' perspectives of care coordination for asthma control between primary and specialized levels of public health care systems in Ecuador and Brazil during the COVID-19 pandemic.

METHODOLOGY

Study context

The present study is part of a study ("Asthma Attacks Causes and Prevention Study in Urban Latin America (ATTACK)") developed since 2019 in collaboration with partners from the UK, Brazil, and Ecuador. The study seeks to understand better the causality of asthma attacks and of their recurrence and optimize strategies to improve asthma control and prevent asthma attacks in Latin America (LA).

Study design

A qualitative research design will be used to explore health workers' perspective in asthma control to characterize and compare health care networks in Ecuador and Brazil during the COVID-19 pandemic. Data will be analysed using a narrative approach focusing on the participants perception, whereby the researcher and the participant develop jointly results from an interactive conversation (18,19). Narrative theory aims to understand the succession of facts, situations, phenomena, processes, and events where thoughts, feelings, emotions, and interactions are involved, through the experiences told by those who experienced them (20). The study will be conducted between March 2021 and December 2021.

Theoretical framework

For peer review only

The present study is based on the theoretical framework of Integrated Health Care Networks (IHCN) (21). Health systems must be understood as IHCN, defined as networks of organizations that provide or make arrangements to provide equitable and integrated health services to defined populations that are accountable for their clinical and economic impacts as well as the health of the populations they serve (21). Among the IHCN objectives we can distinguish two groups: 1) Intermediate objectives: a) access, b) care coordination, c) continuous care; 2) Final objectives: a) equity in access and b) efficiency.

In the present study we focus our efforts in the analysis of types of Care Coordination, represented by clinical information coordination, coordination of clinical management and administrative coordination (22).

Clinical information coordination. This focuses on referrals between care levels and use of information from previous episodes and biopsychosocial situations relevant to the current patient consult. The way in which information is transmitted between healthcare professionals affects how current events are linked to previous events and how the current consult is thus adapted to the needs of the patient. For this study, this will be divided into two dimensions: clinical and psychosocial information referral where attributes consisted of documents for the referral, agile and timely access to information, pertinent content of the information and registry of the information by professionals, and the use of the information whose attributes are: transfer of information from the consultation and the incorporation of information into the clinical practice (22).

Coordination of clinical management. The provision of healthcare in a sequential and complementary way, with a clinical management plan shared by the different care levels and participating services. It is defined by three dimensions, Care coherence (similar approximations among the professionals from different levels), Accessibility among levels (provision without interruptions) and Accurate follow up of the patient among levels (in the transitions from one care level to another) (22). The first dimension being the follow up of the patient that includes attributes such as existence of a physician responsible for patient follow up, communication with those responsible for making the referral and having a follow-up consult after the referral. The second dimension, accessibility among levels, focuses on the provision of attention without

For peer review only

interruption, and the third dimension, care consistency (or coherence), that centres on attributes like: shared objectives, treatment concordance between primary and specialized labels, patient transference consistent with the diagnosis and the duplication of exams, dates and medication.

Administrative coordination. This is defined as the coordination of patient's access along the care continuum according to needs. It works with the administrative circuits dimension, where attributes such as existence of mechanisms for the administrative circuit, administrative referral of the patient to a suitable unit and previous programming of consults, exams and treatment, are studied. The importance of HCC among health system levels constitutes a strategy to achieve continuous care, reduce costs and improve quality of care. The perception of the information dimensions and management of the HCC relies on factors of the professionals, existence and use of coordination mechanisms and organizational factors (22). The theoretical framework is shown in figure 1.

Figure 1. Theoretical Framework for Health Care Coordination. The Theoretical Framework is shown by type of coordination and attributes and dimensions of each.

Study area

The study will be conducted in three cities in Ecuador and one city in Brazil. Ecuador is an upper-middle-income-country with a per capita income of $6110. In 2009 the Integral Public Health Network (IPHN) became a Constitutional mandate, aiming to develop collaborations between private and public sectors. The Public institutions offer health care services to the entire population and are divided into different levels of care and distributed geographically (23). Social security health institutions offer services only to the affiliated salaried population. The private sector includes for-profit entities (hospitals, clinics, dispensaries, doctor's offices, pharmacies, and prepaid health insurance companies) which are generally located in the main cities of the country. Quito, the capital of Ecuador and one of the cities where the study will be conducted, is located in the Andean region at an altitude of 2,800 meters, is one of the most populous Ecuadorian urban area with 2’781,641 inhabitants and has greater socioeconomic and health indicators than the national average. The two other cities in the study are: Cuenca, located at altitude of 2550 meters with a population of 329,928

For peer review only

inhabitants, and Portoviejo located at altitude of 53 meters with a population of 206,682 inhabitants (24–26). Public health care in Ecuadorian cities is divided geographically into sanitary districts: Quito with nine Cuenca with two, and Portoviejo with one. Districts chosen for study were 17D02, 01D01, and 13D01 in Quito, Cuenca, and Portoviejo, respectively.

Brazil is an upper-middle-income-country with a per capita income of $9130 (24). The Brazilian health system has been characterized by the principle of universal healthcare access mandated by its constitution since 1988. The Unique Health System of Brazil (SUS) has different levels of care including primary and specialized in which the public and private sector participate, and is organized hierarchically and geographically with the aim of supporting SUS to the benefit of the population (27). The municipality of Salvador, capital of the state of Bahia, is the most populous in Northeast Brazilian with a population of 2,953,986 inhabitants living in the urban area (28). The organization of public health care in Salvador is territorial and divided into 12 Sanitary Districts. In Salvador, the Railway District was chosen.

Participants: Study subjects

Family doctors, nurses, specialists, and public health representatives working in primary, secondary and tertiary care levels involved in the care of patients with asthma will be invited to participate. We will recruit a minimum of 20 participants per country to achieve speech saturation. Inclusion criteria are: a) Ecuadorian and Brazilian health professionals with at least 6 months experience; b) public health representatives with a wide range of experience 3) managers with senior positions (i.e directors) at levels of health care. A flow diagram for participant recruitment is shown in Figure 2.

Figure 2. Flow diagram for recruitment showing how health actors become participants.

Data Collection, instruments, and procedures

Data Collection

In-depth interviews will be used for data collection to explore health workers' experiences and views on HCC for asthma. Contacts due to the COVID-19 pandemic will be via cell phone, WhatsApp or email. Study documentation shared with the

For peer review only

participants are: information letter, informed consent for recording of video calls and for participation in the study, and the Participant information sheet.

Participants will be asked to openly describe their experiences and vision about care coordination. The interview will be conducted by two persons from the study team composed of an interviewer and an observer. Interviewers will use a semi-structured guideline including the following topics:

1. Asthma management in primary and specialized care levels.

2. Availability and use of asthma guidelines or care protocols by health workers

3. Care Coordination for asthma patients between primary and specialized care levels

4. Transfer protocols between primary, secondary, and tertiary care levels

5. Understanding of HCC and Integrated Health Network

6. Strategies, interventions, and instruments in HCC

7. Changes and adaptations of asthma control during the COVID-19 context.

Procedure

Procedures are planned as follows: a) informants will be contacted by gatekeepers in each city; b) an introductory letter (and informed consent forms) will be provided to each informant explaining the study; c) if the participant agrees to be part in the study and signs consent , it the following will be done; d) each interview, researcher and participant will be assigned an alphanumeric unique identifier code to ensure confidentiality; e) each interview will last approximately 50 minutes; and f) interviews will be audio-recorded and transcribed.

Data Analysis tools and procedures

This study is based on narrative analysis of manifest content through a priori categories and using constant comparisons between the speech of each of the defined profiles as well as emerging categories. Narrative analysis will be segmented by case, type of actor and topic. The content of each interview will be read by the researcher, identifying significant fragments, classified by type of informant, with the purpose of identifying similarities and differences. We will use descriptive data regarding the Health Systems of Brazil and Ecuador to establish comparisons between the countries

For peer review only

(comparative table). In this study we will produce triangulations among informants, researchers, and the theoretical framework. Analysis will use NVivo (Neu, QSR International).

The triangulation process will allow us to work with three different levels of analysis. At the first level the significant phrases will be codified using the theoretical framework from the participants transcript interviews, aiming to saturate speech. At the second level two different researchers from each country will analyse the chosen significant phrases and decide if the significant phrases correspond to the categories or not. After this process, the second level and first level researchers meet up in a discussion of the results before moving on to the third level. At the third level with the consolidated information from the first two levels, the experts make an analysis of the significant phrases using the theoretical framework and comments made at other levels before deciding if a category is saturated. The information is once again discussed between the three levels before accepting it as a significant phrase.

Extensive training was provided to the researchers participating in the study at all levels aiming to standardize concepts and procedures.

Patient and Public Involvement

No patient involved.

ETHICS AND DISSEMINATION

The protocol was approved by the Ethics Committees of the Hospital General Docente Calderón (CEISH-HGDC 2019-001) in Ecuador and Faculdade de Medicina da Bahia da Universidade Federal da Bahia (CAAE: 04057518.0.0000.5577) in Brazil. The study will be done in accordance with the guidelines of the World Medical Association and the Declaration of Helsinki.

This study forms a part of the NIHR Global Health Research Group "Asthma Attacks Causes and Prevention Study in Urban Latin America". We acknowledge that all participants must be available and be capable of participating in the study and that there is a small chance that the interviewees could create a role of power among the interviewers if the purpose of the research is not understood. To tackle these issues, we will inform all participants about the purpose of the research and will obtain written informed consent from each participant. Besides, designing an interview guide, we

For peer review only

have had to consider how to formulate questions in a way that minimizes feelings of discomfort, judgement and criticism among participants.

Our findings will be shared first with the participants of the study and researchers in collaborating institutions. The result will be presented as practical recommendations in each country (Ecuador and Brazil) at policy briefings and forums of healthcare professionals. Our results will also be presented at seminars, academic conferences and in peer-review scientific articles.

Acknowledgments

We acknowledge the contribution institutions that have provided us their financial support.

Author Contributions

All the authors agree with the manuscript's contents. NRS, EA, ARS, ABO, MRF contributed to the design of the study. MJC, EG, CB, ALB will conduct the interviews, and coded the data. NRS, MRF and GP will advise throughout all phases of the study. AR, AC, and PC will be provided input. NRS, EA, ARS, ABO and MRF will conduct the coding schema and all phases of analysis. All authors contributed to the write-up.

Funding. This study forms part of the Asthma ATTACK study and was funded through the NIHR Global Health Research Group at St George's University of London by the National Institute of Health Research, UK, using Official Development Assistance (ODA) funding.

Grant number: 17/63/62.

Competing interests. None declared.

Patient consent for publication. Not required

Provenance and peer review. Not commissioned.

Open access. This is an open access article distributed in accordance with the Creative Commons Attribution Non-Commercial (CC BY-NC 4.0) license, which permits others to distribute, remix, adapt, build upon this work non-commercially, and license their derivative works on different terms, provided the original work is properly

For peer review only

cited, appropriate credit is given, any changes made indicated, and the use is non-commercial. See: http://creativecommons.org/licenses/by-nc/4.0/.

Data statement

Technical appendix, statistical code, and dataset available from the Dryad repository, DOI: ORCID iD Maria Cisneros-Caceres **https://orcid.org/0000-0001-5996-5147**

Word Count: 2804

REFERENCES

1. van de Veen, W; Akdis, M (2014). Mechanisms of immune regulation in allergy. *EAACI Global Atlas of Allergy.* Zurich: European Academy of Allergy and Clinical Immunology, 90-91. DOI: https://doi.org/10.5167/uzh-140934

2. Ardura-Garcia C, Stolbrink M, Zaidi S, et al. Predictors of repeated acute hospital attendance for asthma in children: A systematic review and meta-analysis. *Pediatr Pulmonol.* 2018 Sep;53(9):1179–92. DOI: 10.1002/ppul.24068

3. Asher I, Pearce N. Global burden of asthma among children. *Int J Tuberc Lung Dis Off J Int Union Tuberc Lung Dis*. 2014 Nov;18(11):1269–78. DOI: 10.5588/ijtld.14.0170

4. Pearce N, Aït-Khaled N, Beasley R, et al. Worldwide trends in the prevalence of asthma symptoms: phase III of the International Study of Asthma and Allergies in Childhood (ISAAC). *Thorax.* 2007 Sep 1;62(9):758–66. DOI: 10.1136/thx.2006.070169

5. Braman SS. The global burden of asthma. *Chest.* 2006 Jul;130(1 Suppl):4S-12S. DOI: 10.1378/chest.130.1_suppl.4S

6. The Global Asthma Report 2018 [Internet]. [cited 2021 Apr 22]. Available from: http://globalasthmareport.org/

7. Janevic MR, Stoll S, Wilkin M, et al. Pediatric Asthma Care Coordination in Underserved Communities: A Quasiexperimental Study. *Am J Public Health*. 2016;106(11):2012–8. doi: 10.2105/AJPH.2016.303373

For peer review only

8. Redes Integradas de Servicios de Salud. OPS.pdf [Internet]. [cited 2020 Oct 13]. Available from: https://www.paho.org/uru/index.php?option=com_docman&view=download&alias=145-redes-integradas-de-servicios-de-salud-aps-n4&category_slug=publicaciones-sistemas-y-servicios-de-salud&Itemid=307

9. Haw J, Cunningham S, O'Doherty KC.J, et al. Epistemic tensions between people living with asthma and healthcare professionals in clinical encounters. *Soc Sci Med* 1982. 2018; May 1;208:34–40. DOI: 10.1016/j.socscimed.2018.04.054

10. Hirshon JM, Risko N, Calvello EJB, et al. Health systems and services: the role of acute care. *Bull World Health Organ*. 2013 May 1;91(5):386–8. DOI: 10.2471/BLT.12.112664

11. Vázquez M-L, Vargas I, Garcia-Subirats I, et al. Doctors' experience of coordination across care levels and associated factors. A cross-sectional study in public healthcare networks of six Latin American countries. *Soc Sci Med.* 2017 Jun 1;182:10–9. DOI: 10.1016/j.socscimed.2017.04.001

12. Souza-Machado C, Souza-Machado A, Franco R, et al. Rapid reduction in hospitalisations after an intervention to manage severe asthma. *Eur Respir J.* 2010 Mar;35(3):515–21. DOI: 10.1183/09031936.00101009

13. Soto-Martínez M, Avila L, Soto N, et al. Trends in hospitalizations and mortality from asthma in Costa Rica over a 12- to 15-year period. *J Allergy Clin Immunol Pract.* 2014 Feb;2(1):85–90. DOI: 10.1016/j.jaip.2013.09.010

14. Neffen H, Fritscher C, Schacht FC, et al. Asthma control in Latin America: The Asthma Insights and Reality in Latin America (AIRLA) survey. *Rev Panam Salud Publica Pan Am J Public Health*. 2005 Mar;17(3):191–7. DOI: 10.1590/s1020-49892005000300007

15. Solé D, Aranda CS, Wandalsen GF. Asthma: epidemiology of disease control in Latin America – short review. *Asthma Res Pract.* 2017 May 11;3(1):4. DOI: https://doi.org/10.1186/s40733-017-0032-3

For peer review only

16. La OMS caracteriza a COVID-19 como una pandemia - OPS/OMS | Organización Panamericana de la Salud [Internet]. [cited 2021 Apr 22]. Available from: https://www.paho.org/es/noticias/11-3-2020-oms-caracteriza-covid-19-como-pandemia

17. Ministerio de Salud Pública de Ecuador: ratifica la actual vigencia de la Emergencia Sanitaria – Ministerio de Salud Pública [Internet]. [cited 2021 Apr 22]. Available from: https://www.salud.gob.ec/msp-ratifica-la-actual-vigencia-de-la-emergencia-sanitaria/

18. Bastos LC, Biar L de A, Bastos LC, et al. Análise de narrativa e práticas de entendimento da vida social. *DELTA Doc Estud Em Lingüíst Teórica E Apl.* 2015 Aug;31(SPE):97–126. DOI: http://dx.doi.org/10.1590/0102-445083363903760077

19. Castellanos MEP. The narrative in qualitative research in health. *Ciênc Amp Saúde Coletiva.* 2014 Apr;19(4):1065–76. DOI: 10.1590/1413-81232014194.12052013

20. Hernández-Sampieri, R. & Mendoza, C (2018). Metodología de la investigación. Las rutas cuantitativa, cualitativa y mixta | RUDICS [Internet]. [cited 2020 Oct 13]. Available from: http://virtual.cuautitlan.unam.mx/rudics/?p=2612

21. Shortell SM, Anderson DA, Gillies RR, et al. Building integrated systems--the holographic organization. *Health Forum J*. 1993 Apr;36(2):20–6. PMID: 10124166

22. Terraza Núñez R, Vargas Lorenzo I, Vázquez Navarrete ML. La coordinación entre niveles asistenciales: una sistematización de sus instrumentos y medidas. *Gac Sanit.* 2006 Nov 1;20(6):485–95.

23. Manual MAIS-MSP12.12.12 [Internet]. [cited 2021 Apr 22]. Available from: http://instituciones.msp.gob.ec/somossalud/images/documentos/guia/Manual_MAIS-MSP12.12.12.pdfhttps://es.scribd.com/document/338645477/Manual-MAIS-MSP12-12-12-pdf

24. GNI per capita, Atlas method (current US$) | Data [Internet]. [cited 2020 Oct 13]. Available from: https://data.worldbank.org/indicator/NY.GNP.PCAP.CD

For peer review only

25. Instituto Nacional de Estadística y Censos de Ecuador [Internet]. [cited 2020 Oct 13]. Available from: https://www.ecuadorencifras.gob.ec/institucional/home/

26. Lucio R, Villacrés N, Henríquez R. Sistema de salud de Ecuador. *Salud Pública México.* 2011;53:12. Avalaible: http://www.redalyc.org/articulo.oa?id=10619779013

27. Paim J, Travassos C, Almeida C, et al. The Brazilian health system: history, advances, and challenges. *Lancet Lond Engl*. 2011 May 21;377(9779):1778–97. DOI: 10.1016/S0140-6736(11)60054-8

28. Salvador (BA) | Cidades e Estados | IBGE [Internet]. [cited 2021 Apr 22]. Available from: https://www.ibge.gov.br/cidades-e-estados/ba/salvador.html

For peer review only

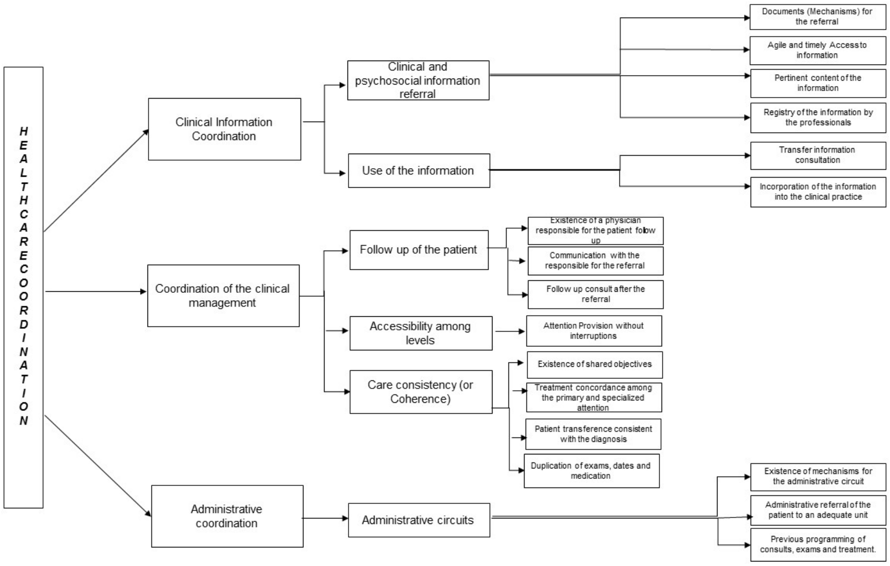

Theoretical Framework for Health Care Coordination. The Theoretical Framework is shown by type of coordination and attributes and dimensions of each.

338x190mm (96 x 96 DPI)

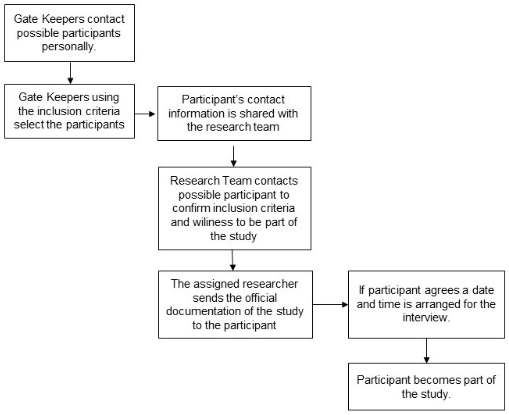

Flow diagram for recruitment showing how health actors become participants.

338x190mm (96 x 96 DPI)

BMJ Open

Health worker's perspectives on asthma care coordination between primary and specialized health care in the COVID-19 pandemic: a protocol for a qualitative study in Ecuador and Brazil.

Journal:	*BMJ Open*
Manuscript ID	bmjopen-2021-052971.R1
Article Type:	Protocol
Date Submitted by the Author:	21-Sep-2021
Complete List of Authors:	Romero, Natalia; Universidad Internacional del Ecuador, School of Medicine; Red Grups d'Amèrica i Àfrica Llatines<br>Cisneros-Caceres, Maria; Universidad Internacional del Ecuador, School of Medicine<br>Granadillo, Emily; Universidad Internacional del Ecuador, School of Medicine<br>Aragao, Erika; Universidade Federal da Bahia<br>Romero-Sandoval, Adriana; Universidad Internacional del Ecuador, School of Basic Sciences; Red Grups d'Amèrica i Àfrica Llatines<br>Barbosa, Carolina; Universidade Federal da Bahia<br>Barreto de Oliveira, Ana Luiza; Universidade Federal da Bahia<br>Rodriguez, Alejandro ; Universidad Internacional del Ecuador, School of Medicine<br>Pinheiro, Gabriela; Fundação ProAR,<br>Cruz, Alvaro; Universidade Federal da Bahia<br>Cooper, Philip; St George's University of London, Institute of Infection and Immunity; Universidad Internacional del Ecuador, School of Medicine<br>Ferreira da Silva, Maria Rejane; UPE; Red Grups d'Amèrica i Àfrica Llatines
**Primary Subject Heading**:	Health services research
Secondary Subject Heading:	Respiratory medicine, Medical management
Keywords:	Asthma < THORACIC MEDICINE, COVID-19, Quality in health care < HEALTH SERVICES ADMINISTRATION & MANAGEMENT, HEALTH SERVICES ADMINISTRATION & MANAGEMENT, Organisation of health services < HEALTH SERVICES ADMINISTRATION & MANAGEMENT

SCHOLARONE™
Manuscripts

I, the Submitting Author has the right to grant and does grant on behalf of all authors of the Work (as defined in the below author licence), an exclusive licence and/or a non-exclusive licence for contributions from authors who are: i) UK Crown employees; ii) where BMJ has agreed a CC-BY licence shall apply, and/or iii) in accordance with the terms applicable for US Federal Government officers or employees acting as part of their official duties; on a worldwide, perpetual, irrevocable, royalty-free basis to BMJ Publishing Group Ltd ("BMJ") its licensees and where the relevant Journal is co-owned by BMJ to the co-owners of the Journal, to publish the Work in this journal and any other BMJ products and to exploit all rights, as set out in our licence.

The Submitting Author accepts and understands that any supply made under these terms is made by BMJ to the Submitting Author unless you are acting as an employee on behalf of your employer or a postgraduate student of an affiliated institution which is paying any applicable article publishing charge ("APC") for Open Access articles. Where the Submitting Author wishes to make the Work available on an Open Access basis (and intends to pay the relevant APC), the terms of reuse of such Open Access shall be governed by a Creative Commons licence – details of these licences and which Creative Commons licence will apply to this Work are set out in our licence referred to above.

Other than as permitted in any relevant BMJ Author's Self Archiving Policies, I confirm this Work has not been accepted for publication elsewhere, is not being considered for publication elsewhere and does not duplicate material already published. I confirm all authors consent to publication of this Work and authorise the granting of this licence.

For peer review only

Health worker's perspectives on asthma care coordination between primary and specialized health care in the COVID-19 pandemic: a protocol for a qualitative study in Ecuador and Brazil.

Natalia Cristina Romero Sandoval [1,7] María José Cisneros-Caceres[1], Emily Granadillo[1], Erika Aragao[2], Adriana Romero Sandoval[3,7], Carolina Barbosa[4], Ana Luiza Barreto de Oliveira[4], Alejandro Rodríguez[1], Gabriela Pimentel Pinheiro[4], Álvaro A. Cruz[4], Philip Cooper[1,5], Maria Rejane Ferreira da Silva[6 7]

[1]. School of Medicine, Universidad Internacional del Ecuador, Quito, Ecuador

[2]. Instituto de Saúde Coletiva - Universidade Federal da Bahia, Salvador de Bahia – Brazil

[3]. School of Basic Sciences, Universidad Internacional del Ecuador, Quito, Ecuador

[4]. Programa para o Controle da Asma na Bahia (ProAr), Faculdade de Medicina, Universidade Federal da Bahia, Salvador de Bahia – Brazil

[5]. Institute of Infection and Immunity, St George's University of London, London, UK.

[6].Universidade de Pernambuco/UPE, Pernambuco – Brazil

[7]. Red Grups d'Amèrica i Àfrica Llatines – GRAAL

NCR nromero@uide.edu.ec

MJC majocc93@gmail.com

EG egranadillo@uide.edu.ec

EA erikapecs@gmail.com

ARS adromero@uide.edu.ec

CB carolinabarbosa.ss@gmail.com

ALB annabarreto12@yahoo.com.br

AR rorodriguezal@uide.edu.ec

GPP gabrielacear@gmail.com

AAC cruz.proar@gmail.com

PC pcooper@sgul.ac.uk

MRF rejane.pernambuco@gmail.com

Corresponding author: Maria Jose Cisneros. Av. Jorge Fernández and Simón Bolívar, Quito, Ecuador. Telf: + 593 2985600

For peer review only

ABSTRACT

Introduction. Asthma is a common long-term disorder and strategies to improve asthma control are still a challenge. Integrated delivery of health systems is critical for effective asthma care: there is limited information on experiences of care coordination for asthma from Latin America, especially on perspectives of health personnel and in the context of the COVID-19 pandemic.

Methods and analysis. This protocol details a qualitative approach to analyse health workers' perspectives of health care coordination for asthma control during COVID-19 pandemic in Ecuador and Brazil, at primary and specialized levels, through in-depth semi-structured interviews using a video communications platform. The analysis will identify knowledge and perspectives based on coordination of clinical information, clinical management, and administrative coordination. Theoretical sampling will be used to obtain approximately equal numbers of women and men within each level of healthcare; data saturation will be used to determine sample size. Transcripts will be analysed using content coding procedures to mark quotations related to major topics and sub-themes included in the interview guide, and narrative analysis will be based on a theoretical framework for health care coordination to identify new themes and sub-themes.

Ethics and dissemination. Ethical approval was obtained from the ethics committees of Hospital General Docente Calderón, Quito, Ecuador, and Universidade Federal da Bahia, Salvador, Brazil. The findings of this study will be disseminated through peer–reviewed articles, conference presentations and condensed summaries for key stakeholders and partners.

Keywords: *Asthma, Coronavirus Infections, Care Coordination, Integrated delivery systems, Health Personnel*

For peer review only

STRENGTHS AND LIMITATIONS OF THIS STUDY

- This qualitative study protocol is the first to focus on the perspectives of healthcare workers on asthma care coordination between primary and specialized levels in the context of the COVID-19 pandemic.
- This study will use in-depth semi-structured interviews of healthcare workers from four cities in Ecuador and Brazil to compare perspectives on asthma care and healthcare system coordination between locations.
- Qualitative interviews will be done over the period of a year during the COVID-19 pandemic that likely will affect coordination of asthma care.
- Although our recruitment method should identify key informants, we could not involve the best representatives from primary and specialized care levels.

For peer review only

INTRODUCTION

Asthma has emerged as a major challenge for health care systems around the world accounting for millions of doctor visits, hundreds of thousands of emergency department visits and hospitalizations, and thousands of deaths [1,2]. Among those who have asthma, children are the most affected group and asthma is now the most common chronic disease of childhood [3]. While our knowledge on asthma has increased, we still have an incomplete understanding of its causes: factors including environmental exposures, changes in lifestyles and host genetics are likely to be important [1].

There are wide variations in the prevalence of asthma symptoms between countries [4]. However, trends in asthma mortality and hospitalization have shown a progressive reduction or stabilization, especially in high income countries (HICs), where asthma prevalence reached epidemic levels four decades ago [5]. Presently, although the prevalence of asthma has stabilized in HICs, it appears to be increasing in some low and middle-income countries (LMICs), regions that account for more than 80% of asthma deaths worldwide. Nonetheless, many cases of severe asthma and asthma deaths are preventable through optimal management using medications to relieve and control the disease and improvements in health care coordination (HCC) [1,6].

HCC has been shown to be a promising strategy to improve asthma management and control in HICs: improved care coordination results in reduced asthma symptoms and urgent health care utilization which is cost-effective [7]. However, in LMICs health care coordination remains a challenge for asthma patients because most of the health systems in these regions are characterized by fragmentary organization and a lack of resources [8]. Additionally, medical therapy is often hindered by fragmented disease-specific approaches to management (fostered by single-disease guidelines), use of acute care to manage chronic diseases, and inadequate integration of care across multiple levels, especially from hospital to home [9,10].

In Latin America (LA), HCC has been characterized by a reconstruction of the self-identity concept [11]. There are few reports of HCC implementation [12,13], while asthma management and control are uncoordinated in most health systems, showing high rates of acute exacerbations, and use of urgent care with high hospitalization rates [14,15].

The outbreak of infections with the novel coronavirus, SARS-CoV-2, in Central China, in December 2019 marked the beginning of the COVID-19 pandemic [16]. Measures to reduce viral transmission were adopted in Ecuador and Brazil from March 2020 with enforced social isolation and restricted movement at local or national levels interfering with opportunities for interactions with healthcare. Although lockdowns had been lifted by the second half of 2020,

For peer review only

there was still limited access to emergency rooms and outpatient care at primary and specialized levels for non-COVID-19 consults [17], a situation that continued into the first quarter of 2021.

This protocol details the methodological and analytical considerations using a qualitative approach in a study designed to understand and characterize healthcare workers' perspectives of care coordination for asthma control between primary and specialized levels of public health care systems in Ecuador and Brazil during the COVID-19 pandemic.

Based on the characteristics of the qualitative research, we are flexible with the completion date for the study. Still holding in-depth interviews as well as data collecting until we reach theoretical saturation, expecting to have results by the first quarter of 2022.

METHODOLOGY

Study context

The present study is part of a study ("Asthma Attacks Causes and Prevention Study in Urban Latin America (ATTACK)") developed in 2019 in collaboration with partners from the UK, Brazil, and Ecuador. The study seeks to understand better the causality of asthma attacks and of their recurrence and optimize strategies to improve asthma control and prevent asthma attacks in Latin America (LA).

Study design

A qualitative research design will be used to explore health workers' perspective in asthma control to characterize and compare health care networks in Ecuador and Brazil during the COVID-19 pandemic. Data will be analysed using a narrative approach focusing on the participants perception, whereby the researcher and the participant develop jointly results from an interactive conversation [18,19]. Narrative theory aims to understand the succession of facts, situations, phenomena, processes, and events where thoughts, feelings, emotions, and interactions are involved, through the experiences told by those who experienced them [20].

Theoretical framework

The present study is based on the theoretical framework of Integrated Health Care Networks (IHCN) [21]. Health systems must be understood as IHCN, defined as networks of organizations that provide or make arrangements to provide equitable and integrated health services to defined populations that are accountable for their clinical and economic impacts as well as the health of the populations they serve [21]. Among the IHCN objectives we can

For peer review only

distinguish two groups: 1) Intermediate objectives: a) access, b) care coordination, c) continuous care; 2) Final objectives: a) equity in access and b) efficiency.

In the present study we focus our efforts on the analysis of types of Care Coordination, represented by clinical information coordination, coordination of clinical management and administrative coordination [22].

Clinical information coordination. This focuses on referrals between care levels and use of information from previous episodes and biopsychosocial situations relevant to the current patient consultation. The way in which information is transmitted between healthcare professionals affects how current events are linked to previous events and how the current consult is thus adapted to the needs of the patient. For this study, this will be divided into two dimensions: clinical and psychosocial information referral where attributes consisted of documents for the referral, agile and timely access to information, pertinent content of the information and registry of the information by professionals, and the use of the information whose attributes are: transfer of information from the consultation and the incorporation of information into the clinical practice [22].

Coordination of clinical management. The provision of healthcare in a sequential and complementary way, with a clinical management plan shared by the different care levels and participating services. It is defined by three dimensions, Care coherence (similar approximations among the professionals from different levels), Accessibility among levels (provision without interruptions) and Accurate follow up of the patient among levels (in the transitions from one care level to another) [22]. The first dimension being the follow up of the patient that includes attributes such as existence of a physician responsible for patient follow up, communication with those responsible for making the referral and having a follow-up consult after the referral. The second dimension, accessibility among levels, focuses on the provision of attention without interruption, and the third dimension, care consistency (or coherence), that centres on attributes like: shared objectives, treatment concordance between primary and specialized labels, patient transference consistent with the diagnosis and the duplication of exams, dates, and medication.

Administrative coordination. This is defined as the coordination of patient's access along the care continuum according to needs. It works with the administrative circuits dimension, where attributes such as existence of mechanisms for the administrative circuit, administrative referral of the patient to a suitable unit and previous programming of consults, exams and treatment, are studied. The importance of HCC among health system levels constitutes a strategy to achieve continuous care, reduce costs and improve quality of care. The perception of the information dimensions and management of the HCC relies on factors of the

For peer review only

professionals, existence and use of coordination mechanisms and organizational factors [22]. The theoretical framework is shown in figure 1.

Figure 1

Study area

The study will be conducted in three cities in Ecuador and one city in Brazil. Ecuador is an upper-middle-income-country with a per capita income of $6110. According to the World Health Organization's Global Health Workforce Statistics, the physicians per 1000 people for Ecuador and Brazil are around 2.2, and the nurses 10.1 for Brazil and 2.5 for Ecuador [23, 24]. In 2009 the Integral Public Health Network (IPHN) became a Constitutional mandate, aiming to develop collaborations between private and public sectors. The Public institutions offer health care services to the entire population and are divided into different levels of care and distributed geographically [25]. Social security health institutions offer services only to the affiliated salaried population. The private sector includes for-profit entities (hospitals, clinics, dispensaries, doctor's offices, pharmacies, and prepaid health insurance companies) which are generally located in the main cities of the country. Quito, the capital of Ecuador and one of the cities where the study will be conducted, is located in the Andean region at an altitude of 2,800 meters, is one of the most populous Ecuadorian urban area with 2’781,641 inhabitants and has greater socioeconomic and health indicators than the national average. The two other cities in the study are: Cuenca, located at altitude of 2550 meters with a population of 329,928 inhabitants, and Portoviejo located at altitude of 53 meters with a population of 206,682 inhabitants [26–28]. Public health care in Ecuadorian cities is divided geographically into sanitary districts: Quito with nine Cuenca with two, and Portoviejo with one. Districts chosen for study were 17D02, 01D01, and 13D01 in Quito, Cuenca, and Portoviejo, respectively.

Brazil is an upper-middle-income-country with a per capita income of $9130 [26]. The Brazilian health system has been characterized by the principle of universal healthcare access mandated by its constitution since 1988. The Unique Health System of Brazil (SUS) has different levels of care including primary and specialized in which the public and private sector participate and is organized hierarchically and geographically with the aim of supporting SUS to the benefit of the population [29]. The municipality of Salvador, capital of the state of Bahia, is the most populous in Northeast Brazil with a population of 2,953,986 inhabitants living in the urban area [30]. The organization of public health care in Salvador is territorial and divided into 12 Sanitary Districts. In Salvador, the Railway District was chosen.

For peer review only

Both in Ecuador and Brazil, the health care models include general principles of distribution of tasks between the community level, first level and the specialist level, through referral and counter-referral, minimizing duplication of functions and competition between levels. Although guidelines are provided by each national health ministry, we would like to understand better how this coordination between levels functions using a chronic disease such as asthma as a model condition.

Participants: Study subjects

Health actors are defined as individuals or groups with an interest in the health system [31]. Family doctors, nurses, specialists, and public health representatives working in primary, secondary, and tertiary care levels involved in the care of patients with asthma will be invited to participate. Gatekeepers which are health professionals that work on a regular basis with asthma patients and within the healthcare system in both countries, will be responsible for the first contact. We will recruit a minimum of 20 participants per country to achieve speech saturation. Inclusion criteria are a) Ecuadorian and Brazilian health professionals with at least 6 months experience; b) public health representatives with a wide range of experience 3) managers with senior positions (i.e directors) at levels of health care. A flow diagram for participant recruitment is shown in Figure 2.

Figure 2

Patient and Public Involvement

No patient involved

Data Collection, instruments, and procedures

Data Collection

In-depth interviews will be used for data collection to explore health workers' experiences and views on HCC for asthma. Contacts due to the COVID-19 pandemic will be via cell phone, WhatsApp, or email. Study documentation shared with the participants are an information letter, informed consent for recording of video calls and for participation in the study, and the Participant information sheet.

Participants will be asked to openly describe their experiences and vision about care coordination. The interview will be conducted by two persons from the study team composed of an interviewer and an observer. Interviewers will use a semi-structured guideline including the following topics:

For peer review only

1. Asthma management in primary and specialized care levels.

2. Availability and use of asthma guidelines or care protocols by health workers

3. Care Coordination for asthma patients between primary and specialized care levels

4. Transfer protocols between primary, secondary, and tertiary care levels

5. Understanding of HCC and Integrated Health Network

6. Strategies, interventions, and instruments in HCC

7. Changes and adaptations of asthma control during the COVID-19 context.

Procedure

Procedures are planned as follows: a) informants will be contacted by gatekeepers in each city; b) an introductory letter (and informed consent forms) will be provided to each informant explaining the study; c) if the participant agrees to be part in the study and signs consent , it the following will be done; d) each interview, researcher and participant will be assigned an alphanumeric unique identifier code to ensure confidentiality; e) each interview will last approximately 50 minutes; and f) interviews will be audio-recorded and transcribed.

Data Analysis tools and procedures

This study is based on narrative analysis of manifest content through a priori categories and using constant comparisons between the speech of each of the defined profiles as well as emerging categories. Narrative analysis will be segmented by case, type of actor and topic. The content of each interview will be read by the researcher, identifying significant fragments, classified by type of informant, with the purpose of identifying similarities and differences. We will use descriptive data regarding the Health Systems of Brazil and Ecuador to establish comparisons between the countries (comparative table). In this study we will produce triangulations among informants, researchers, and the theoretical framework. Analysis will use NVivo (Neu, QSR International).

The triangulation process will allow us to work with three different levels of analysis. At the first level the significant phrases will be codified using the theoretical framework from the participants transcript interviews, aiming to saturate speech. At the second level two different researchers from each country will analyse the chosen significant phrases and decide if the significant phrases correspond to the categories or not. After this process, the second level and first level researchers meet up in a discussion of the results before moving on to the third level. At the third level with the consolidated information from the first two levels, the experts make an analysis of the significant phrases using the theoretical framework and comments

For peer review only

made at other levels before deciding if a category is saturated. The information is once again discussed between the three levels before accepting it as a significant phrase.

Extensive training was provided to the researchers participating in the study at all levels aiming to standardize concepts and procedures.

For the construction of the analysis and reporting of results we will follow the recommendations SRQR guideline [32].

ETHICS AND DISSEMINATION

The protocol was approved by the Ethics Committees of the Hospital General Docente Calderón (CEISH-HGDC 2019-001) in Ecuador and Faculdade de Medicina da Bahia da Universidade Federal da Bahia (CAAE: 04057518.0.0000.5577) in Brazil. The study will be done in accordance with the guidelines of the World Medical Association and the Declaration of Helsinki.

This study forms a part of the NIHR Global Health Research Group "Asthma Attacks Causes and Prevention Study in Urban Latin America". We acknowledge that all participants must be available and be capable of participating in the study and that there is a small chance that the interviewees could create a role of power among the interviewers if the purpose of the research is not understood. To tackle these issues, we will inform all participants about the purpose of the research and will obtain written informed consent from each participant. Besides, designing an interview guide, we have had to consider how to formulate questions in a way that minimizes feelings of discomfort, judgement, and criticism among participants.

Our findings will be shared first with the participants of the study and researchers in collaborating institutions. The result will be presented as practical recommendations in each country (Ecuador and Brazil) at policy briefings and forums of healthcare professionals. Our results will also be presented at seminars, academic conferences and in peer-review scientific articles.

Acknowledgements

Contributors. All the authors agree with the manuscript's contents. NCR, EA, ARS, AAC, MRF contributed to the design of the study. MJC, EG, CB, ALB will conduct the interviews, and coded the data. NCR, MRF and GP will advise throughout all phases of the study. AR, AAC, and PC will be provided input. NCR, EA, ARS, and MRF will conduct the coding schema and all phases of analysis. All authors contributed to the write-up.

For peer review only

Funding. This study forms part of the Asthma ATTACK study and was funded through the NIHR Global Health Research Group at St George's University of London by the National Institute of Health Research, UK (grant 17/63/62) using Official Development Assistance (ODA) funding.

Competing interests. None declared.

Patient consent for publication. Not required

Provenance and peer review. Not commissioned.

Open access. This is an open access article distributed in accordance with the Creative Commons Attribution Non-Commercial (CC BY-NC 4.0) license, which permits others to distribute, remix, adapt, build upon this work non-commercially, and license their derivative works on different terms, provided the original work is properly cited, appropriate credit is given, any changes made indicated, and the use is non-commercial. See: http://creativecommons.org/licenses/by-nc/4.0/.

Word Count: 3396

Figure 1. Theoretical Framework for Health Care Coordination. The Theoretical Framework is shown by type of coordination and attributes and dimensions of each

Figure 2. Flow diagram for recruitment showing how health actors become participants.

For peer review only

REFERENCES

[dataset] [1]. van de Veen, W; Akdis, M (2014). Mechanisms of immune regulation in allergy. *EAACI Global Atlas of Allergy*. Zurich: European Academy of Allergy and Clinical Immunology, 90-91. DOI: https://doi.org/10.5167/uzh-140934

[dataset] [2]. Ardura-Garcia C, Stolbrink M, Zaidi S, et al. Predictors of repeated acute hospital attendance for asthma in children: A systematic review and meta-analysis. *Pediatr Pulmonol.* 2018 Sep;53(9):1179–92. DOI: 10.1002/ppul.24068

[dataset] [3]. Asher I, Pearce N. Global burden of asthma among children. *Int J Tuberc Lung Dis Off J Int Union Tuberc Lung Dis*. 2014 Nov;18(11):1269–78. DOI: 10.5588/ijtld.14.0170

[dataset] [4]. Pearce N, Aït-Khaled N, Beasley R, et al. Worldwide trends in the prevalence of asthma symptoms: phase III of the International Study of Asthma and Allergies in Childhood (ISAAC). *Thorax.* 2007 Sep 1;62(9):758–66. DOI: 10.1136/thx.2006.070169

[dataset] [5]. Braman SS. The global burden of asthma. *Chest.* 2006 Jul;130(1 Suppl):4S-12S. DOI: 10.1378/chest.130.1_suppl.4S

[dataset] [6]. The Global Asthma Report 2018 [Internet]. [cited 2021 Apr 22]. Available from: http://globalasthmareport.org/

[dataset] [7]. Janevic MR, Stoll S, Wilkin M, et al. Pediatric Asthma Care Coordination in Underserved Communities: A Quasiexperimental Study. *Am J Public Health*. 2016;106(11):2012–8. doi: 10.2105/AJPH.2016.303373

[dataset] [8]. Redes Integradas de Servicios de Salud. OPS.pdf [Internet]. [cited 2020 Oct 13]. Available from: https://www.paho.org/uru/index.php?option=com_docman&view=download&alias=145-redes-integradas-de-servicios-de-salud-aps-n4&category_slug=publicaciones-sistemas-y-servicios-de-salud&Itemid=307

For peer review only

[dataset] [9]. Haw J, Cunningham S, O'Doherty KC.J, et al. Epistemic tensions between people living with asthma and healthcare professionals in clinical encounters. *Soc Sci Med* 1982. 2018; May 1;208:34–40. DOI: 10.1016/j.socscimed.2018.04.054

[dataset] [10]. Hirshon JM, Risko N, Calvello EJB, et al. Health systems and services: the role of acute care. *Bull World Health Organ*. 2013 May 1;91(5):386–8. DOI: 10.2471/BLT.12.112664

[dataset] [11]. Vázquez M-L, Vargas I, Garcia-Subirats I, et al. Doctors' experience of coordination across care levels and associated factors. A cross-sectional study in public healthcare networks of six Latin American countries. *Soc Sci Med.* 2017 Jun 1;182:10–9. DOI: 10.1016/j.socscimed.2017.04.001

[dataset] [12]. Souza-Machado C, Souza-Machado A, Franco R, et al. Rapid reduction in hospitalisations after an intervention to manage severe asthma. *Eur Respir J.* 2010 Mar;35(3):515–21. DOI: 10.1183/09031936.00101009

[dataset] [13]. Soto-Martínez M, Avila L, Soto N, et al. Trends in hospitalizations and mortality from asthma in Costa Rica over a 12- to 15-year period. *J Allergy Clin Immunol Pract.* 2014 Feb;2(1):85–90. DOI: 10.1016/j.jaip.2013.09.010

[dataset] [14]. Neffen H, Fritscher C, Schacht FC, et al. Asthma control in Latin America: The Asthma Insights and Reality in Latin America (AIRLA) survey. *Rev Panam Salud Publica Pan Am J Public Health*. 2005 Mar;17(3):191–7. DOI: 10.1590/s1020-49892005000300007

[dataset] [15]. Solé D, Aranda CS, Wandalsen GF. Asthma: epidemiology of disease control in Latin America – short review. *Asthma Res Pract.* 2017 May 11;3(1):4. DOI: https://doi.org/10.1186/s40733-017-0032-3

[dataset] [16]. La OMS caracteriza a COVID-19 como una pandemia - OPS/OMS | Organización Panamericana de la Salud [Internet]. [cited 2021 Apr 22]. Available from: https://www.paho.org/es/noticias/11-3-2020-oms-caracteriza-covid-19-como-pandemia

For peer review only

[dataset] [17]. Ministerio de Salud Pública de Ecuador: ratifica la actual vigencia de la Emergencia Sanitaria – Ministerio de Salud Pública [Internet]. [cited 2021 Apr 22]. Available from: https://www.salud.gob.ec/msp-ratifica-la-actual-vigencia-de-la-emergencia-sanitaria/

[dataset] [18]. Bastos LC, Biar L de A, Bastos LC, et al. Análise de narrativa e práticas de entendimento da vida social. *DELTA Doc Estud Em Lingüíst Teórica E Apl.* 2015 Aug;31(SPE):97–126. DOI: http://dx.doi.org/10.1590/0102-445083363903760077

[dataset] [19]. Castellanos MEP. The narrative in qualitative research in health. *Ciênc Amp Saúde Coletiva.* 2014 Apr;19(4):1065–76. DOI: 10.1590/1413-81232014194.12052013

[dataset] [20]. Hernández-Sampieri, R. & Mendoza, C (2018). Metodología de la investigación. Las rutas cuantitativa, cualitativa y mixta | RUDICS [Internet]. [cited 2020 Oct 13]. Available from: http://virtual.cuautitlan.unam.mx/rudics/?p=2612

[dataset] [21]. Shortell SM, Anderson DA, Gillies RR, et al. Building integrated systems--the holographic organization. *Health Forum J*. 1993 Apr;36(2):20–6. PMID: 10124166

[dataset] [22]. Terraza Núñez R, Vargas Lorenzo I, Vázquez Navarrete ML. La coordinación entre niveles asistenciales: una sistematización de sus instrumentos y medidas. *Gac Sanit.* 2006 Nov 1;20(6):485–95.

[dataset] [23]. Physicians (per 1,000 people) | Data [Internet]. [cited 2021 Sep 09]. Available from: https://data.worldbank.org/indicator/SH.MED.NUMW.P3

[dataset] [24]. Nurses and midwives (per 1,000 people) | Data [Internet]. [cited 2021 Sep 09]. Available from: https://data.worldbank.org/indicator/SH.MED.PHYS.ZS

[dataset] [25]. Manual MAIS-MSP12.12.12 [Internet]. [cited 2021 Apr 22]. Available from: http://instituciones.msp.gob.ec/somossalud/images/documentos/guia/Manual_M

For peer review only

AIS-MSP12.12.12.pdfhttps://es.scribd.com/document/338645477/Manual-MAIS-MSP12-12-12-pdf

[dataset] [26]. GNI per capita, Atlas method (current US$) | Data [Internet]. [cited 2020 Oct 13]. Available from: https://data.worldbank.org/indicator/NY.GNP.PCAP.CD

[dataset] [27]. Instituto Nacional de Estadística y Censos de Ecuador [Internet]. [cited 2020 Oct 13]. Available from: https://www.ecuadorencifras.gob.ec/institucional/home/

[dataset] [28]. Lucio R, Villacrés N, Henríquez R. Sistema de salud de Ecuador. *Salud Pública México.* 2011;53:12. Avalaible: http://www.redalyc.org/articulo.oa?id=10619779013

[dataset] [29]. Paim J, Travassos C, Almeida C, et al. The Brazilian health system: history, advances, and challenges. *Lancet Lond Engl*. 2011 May 21;377(9779):1778–97. DOI: 10.1016/S0140-6736(11)60054-8

[dataset] [30]. Salvador (BA) | Ciudades e Estados | IBGE [Internet]. [cited 2021 Apr 22]. Available from: https://www.ibge.gov.br/cidades-e-estados/ba/salvador.html

[dataset] [31]. Hoffman, S.J., Cole, C.B. Defining the global health system and systematically mapping its network of actors. *Global Health* 14, 38 (2018). https://doi.org/10.1186/s12992-018-0340-2

[dataset] [32]. O'Brien BC, Harris IB, Beckman TJ, Reed DA, Cook DA. Standards for reporting qualitative research: a synthesis of recommendations. Acad Med. 2014;89(9):1245-1251.

For peer review only

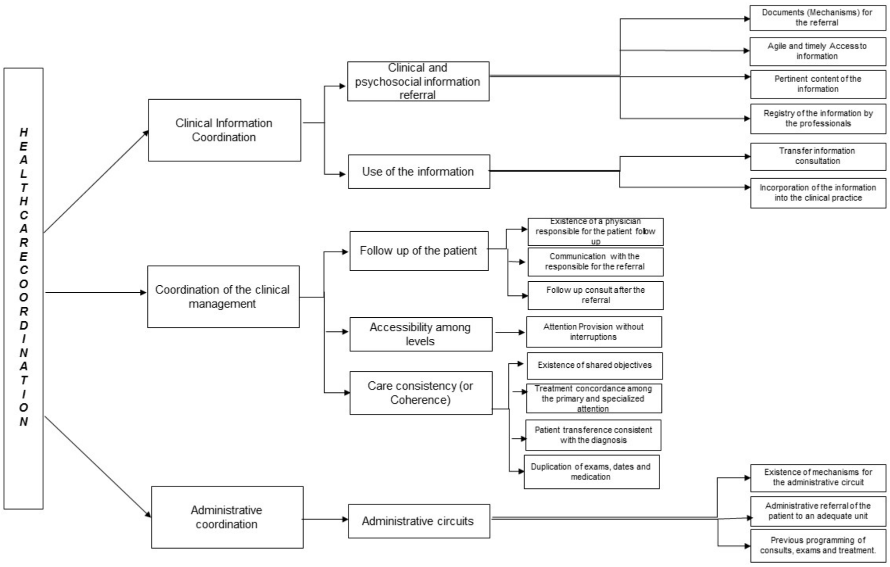

Theoretical Framework for Health Care Coordination. The Theoretical Framework is shown by type of coordination and attributes and dimensions of each.

338x190mm (96 x 96 DPI)

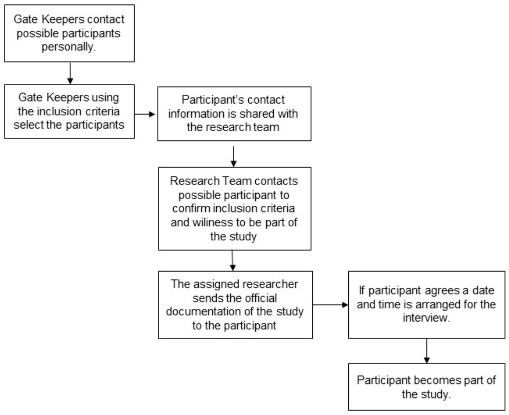

Flow diagram for recruitment showing how health actors become participants.

338x190mm (96 x 96 DPI)
